# Identification of Human and Animal Fecal Contamination after Rainfall in the Han River, Korea

**DOI:** 10.1264/jsme2.ME12187

**Published:** 2013-05-11

**Authors:** Ji Young Kim, Heetae Lee, Jung Eun Lee, Myung-Sub Chung, Gwang Pyo Ko

**Affiliations:** 1 Department of Environmental Health, Graduate School of Public Health, Seoul National University, Kwanak-ro 1, Kwanak-gu, Seoul, 151–752, Republic of Korea; 2 Department of Food Science and Technology, College of Natural Sciences, Chung-Ang University, Ne-ri 72–1, Daeduck-myun, Anseong, Gyeonggi, 456–756, Republic of Korea

**Keywords:** rainfall, fecal contamination, fecal indicator microorganisms, enteric viruses, microbial source tracking

## Abstract

We investigated the effect of rainfall on the levels and sources of microbial contamination in the Han River, Korea. Thirty-four samples were collected at two sampling sites located upstream and downstream in the river from July 2010 to February 2011. Various fecal indicator microorganisms, including total coliform, fecal coliform, *Escherichia coli*, *Enterococcus* spp., somatic and male-specific (F+) coliphage, and four major enteric viruses were analyzed. Rainfall was positively correlated with the levels of fecal coliform and norovirus at both sampling sites. Additionally, rainfall was positively correlated with the levels of total coliform, *E. coli*, *Enterococcus* spp., and F+ coliphage at the upstream site. To identify the source of fecal contamination, microbial source tracking (MST) was conducted using both male-specific (F+) RNA coliphage and the *Enterococcus faecium esp* gene as previously described. Our results clearly indicated that the majority of fecal contamination at the downstream Han River site was from a human source. At the upstream sampling site, contamination from human fecal matter was very limited; however, fecal contamination from non-point animal sources increased following rainfall. In conclusion, our data suggest that rainfall significantly affects the level and source of fecal contamination in the Han River, Korea.

Climate change is an issue of great concern worldwide as over the period between 1900 and 2100, the mean global surface temperature is projected to increase by 1.4 to 5.8°C. In addition to this overall temperature increase, many other aspects of climate, such as humidity and precipitation, are expected to change (11). For example, oceanic evaporation and subsequent precipitation are expected to accelerate, with the average global precipitation expected to increase by >10%, depending on the geographic region and season (10). In Korea, air temperature is reported to increase by 1.5°C, and summer rainfall has significantly increased since the late 1970s (13). Specifically, the Korean Meteorological Administration reported that total precipitation has increased by 7%, whereas the frequency of rainfall has decreased during the 20^th^ century (12). According to the Korean Climate Change Assessment Report of 2010, climate change will result in an increase in heavy rainfall events in Korea (37).

Heavy rainfall events such as storms are considered to be an important factor in determining the fecal contamination of water. During heavy rainfall or snow melt, excess wastewater and runoff can carry contaminated water from non-point sources such that it is directly or indirectly discharged into surface water and seawater (25, 26). Fecal contamination of water can be from natural wildlife as well as anthropogenic sources (30). Contaminated surface or seawater used for shellfish harvesting, food preparation, or recreational purposes can cause water-borne and food-borne diseases (31).

Two major issues should be considered to fully characterize the effects of heavy rainfall on water quality. First, the level of fecal contamination should be addressed. This is typically evaluated using fecal indicator bacteria such as total coliform, fecal coliform, *Escherichia coli*, and *Enterococcus* spp. (29, 32). Additionally, somatic and F+ coliphages have been used as water quality indicators in estuaries, seawater, freshwater, potable water, and wastewater (2).

The second issue to consider is the identification of fecal origin. Microbial source tracking (MST) is a recently developed technique to determine the sources of fecal pollution and pathogens in environmental water. A variety of microorganisms and chemicals have been suggested as targets for MST (29, 32). Among these, male-specific RNA coliphages (F+ RNA coliphages) have been used to successfully identify the source of fecal contamination (20). The genotypes of F+ RNA coliphages are specifically present in either human or animal fecal contamination (5, 20, 35). The enterococcal surface protein (*esp*) gene in *Enterococcus faecium* strains has been suggested as a specific genetic marker of human fecal pollution (15, 28, 36); however, given that no single method has high specificity and sensitivity for identifying the source of fecal contamination, the use of multiple analytical methods has been recommended for MST (29).

During rainfall, the surface water is greatly influenced by point and non-point sources of fecal contamination from farms, parks, and sewage treatment plants. Few studies have addressed the effects and management of water pollution resulting from rainfall and runoff in East Asia, including Korea. The objectives of this study were to (i) examine the effects of rainfall on the levels of fecal indicator microorganisms and enteric viruses in the upper and lower reaches of the Han River, the longest river in Korea, and (ii) identify the source(s) of fecal contamination by MST techniques using F+ RNA coliphages and the *E. faecium esp* gene.

## Materials and Methods

### Sampling locations and time

**Fig. 1:**
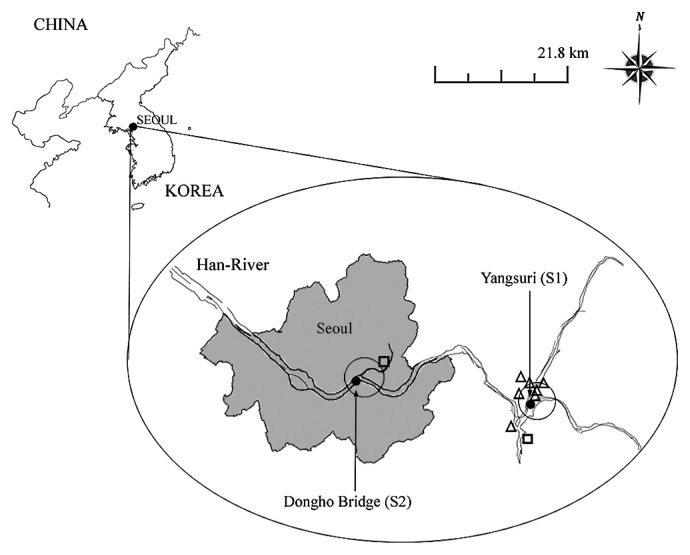
Sampling locations in the Han River (□: sewage treatment plant; Δ: farm).

The Han River, the longest river in Korea, is 469.7 km long with its watershed covering a 26,219 km^2^ area. The river starts from the mountainous Kangwon-Do area and ends in the Yellow Sea on the western side of the Korean peninsula. The river is the primary source for drinking, industrial, and agricultural water for more than 20 million people in the city of Seoul and the surrounding provinces of Kangwon-Do and Kyunggi-Do (14). Two sampling locations, Yangsuri (S1) and Dongho Bridge (S2), along the Han River were chosen for this study (Fig. 1). The upstream S1 location (Yangsuri area) is the confluence site of the Bukhan and Namhan rivers. It is a rural area surrounded by nearby stock farms and farmlands cultivating organic vegetables and fruits such as strawberries around the S1 site. These farms apply composted cow or pig manure to the fields. The upstream region has been designated as a water conservation zone for the nearly 20 million people in metropolitan Seoul. The downstream S2 (Dongho area) site is a highly populated area in the middle of Seoul. Near the S2 sampling site, there is a tributary stream on which a sewage treatment plant is located; however, no discharge from household septic systems into the Han River is allowed. Thirty-four water samples were collected before and after rainfall from July 2010 to February 2011. Rainfall data were obtained from the Korea Meteorological Administration site (http://www.kma.go.kr). Physical-chemical parameters were measured, including pH, temperature, and turbidity.

### Sample collection and analysis of fecal indicator microorganisms

Water samples were collected in 1-L sterile bottles for analysis of fecal indicator microorganisms. The samples were assayed for bacterial fecal indicators (total coliform, fecal coliform, *E. coli*, and *Enterococcus* spp.) using the defined substrate technology test kits, Colilert and Enterolert combined Quanti-tray 2000 (IDEXX Laboratories, Portland, ME). Incubation and enumeration from samples were conducted following the manufacturer’s instructions. In addition to the bacterial fecal indicator, the bacteriophage concentration was analyzed. Each water sample was centrifuged at 3,090×*g* for 20 min to remove large debris. The supernatant was assayed using a single agar layer (SAL) technique following EPA Method 1602 (34). Briefly, to isolate somatic and F+ coliphages from the water sample, 100 mL water sample and 2 mL phage host [*E. coli* CN13 (ATCC 700609) for somatic coliphages and *E. coli* F_amp_ (ATCC 700891) for F+ coliphages] were added to 100 mL of 2×tryptic soy agar (TSA). The concentrations were expressed in plaque-forming units per 100 mL water (PFU 100 mL^−1^). For each assay, either MS2 or phiX174 was used as a positive control of somatic or F+ coliphages.

### Detection of human enteric viruses

#### Sampling and concentration of viruses in water

To detect enteric viruses in water, 10–70 L water was sampled using 5 inches of a positively charged NanoCeram cartridge filter (Argonide, Sanford, FL, USA). After sampling, the cartridge filter was immediately stored at 4°C and analyzed within 6 h of collection. To elute enteric viruses from the cartridge filter, an elution buffer composed of 1.5% beef extract (Difco, Detroit, MI), 0.05 M glycine (pH 9.0), 0.01% Tween80, and 0.001% antiform was used (19). The elution buffer was poured into the housing containing the cartridge filter, left for 15 min, and then continuously re-circulated through the cartridge filter using a peristaltic pump (Masterflex L/S; Cole-Parmer Instrument, Barrington, IL, USA) at a flow rate of 300 mL min^−1^ for 15 min (7). After elution, approximately 600 mL recovered eluate was concentrated to 3–6 mL by polyethylene glycol (PEG) precipitation (19). Each sample was incubated at 4°C for 2 h after mixing with 10% PEG 8000 and 0.2 M NaCl. The pellet was collected by centrifugation at 7,000×*g* at 4°C for 30 min, dissolved in 0.15 M sodium phosphate, and stored at −80°C until further analysis.

#### Real-time PCR analysis of enteric viruses in water

The viral concentrates were filtered (0.45 μm; Millipore, Billerica, MA, USA) prior to viral contamination analysis. Specifically, viral nucleic acid was extracted from 150 μL aliquots of the viral concentrates using a QIAmp Viral RNA mini kit (Qiagen, Valencia, CA, USA). To estimate the concentration of each adenovirus, norovirus (GI, GII), astrovirus, and rotavirus, TaqMan qPCR was performed using the Applied Biosystems 7300 Real-Time PCR (Applied Biosystems, Carlsbad, CA, USA). The reaction mixture (25 μL) for adenovirus contained 2 μL template DNA, 12.5 μL of 2×TaqMan Universal PCR Master Mix (TaqMan Universal PCR Master Mix; Applied Biosystems), 200 nM of each primer, and 50 nM of probe. The PCR conditions were as follows: 2 min at 50°C; 10 min at 95°C; 40 cycles of 95°C for 15 sec and 60°C for 1 min. An AgPath-ID One-Step RT-PCR Kit (Ambion/Applied Biosystems, Piscataway, NJ, USA) was used to conduct the TaqMan qPCR assay of RNA viruses (norovirus, astrovirus, and rotavirus). The RT-PCR reactions for norovirus (GI, GII), astrovirus, and rotavirus were performed in a 25 μL volume containing 12.5 μL of 2×RT-PCR Buffer, 1 μL of 25×RT-PCR Enzyme Mix, 400 nM of each primer, 50 nM of probe, and 2.5 μL RNA. The RT-PCR conditions for norovirus (both GI and GII) were as follows: 10 min at 42°C; 10 min at 95°C; 45 cycles of 95°C for 15 s, and 60°C for 1 min. The thermal cycling conditions for astrovirus and rotavirus were as follows: 45 min at 50°C (30 min at 60°C for rotavirus), followed by 40 cycles of three steps consisting of 30 s at 94°C, 30 s at 50°C, and 30 s at 72°C. To construct quantitative standards for enteric viruses, the viral DNA sequences were amplified using previously reported primers: JHKXF/JHKXR for adenovirus, NNKP1F/NKP1R for norovirus GI, KP2F/NKP2R for norovirus GII, AV1/AV2 for astrovirus, and Rota NVP3-F/Rota NVP3-R for rotavirus (16, 18, 23, 24). The amplified PCR products were cloned into the pGEM-T Easy Vector System (Promega Corporation, Madison, WI, USA) and plasmid DNA was purified using a Labopass plasmid DNA purification kit (Cosmo Genetech Corporation, Seoul, Korea). The plasmid was serially diluted 10-fold in nuclease-free water after determining the concentration using a Nanodrop spectrophotometer ND 1000 (NanoDrop Technologies, Wilmington, DE, USA), and standard curves were generated.

### Microbial source tracking

Microbial source tracking (MST) was performed by genotyping F+ RNA coliphages and determining the presence of the *Enterococcus faecium esp* gene. Approximately 20 plaques were selected using sterile wooden toothpicks from the plaques on the F+ coliphage assay plate. Each selected plaque was enriched and isolated as previously described (20). To distinguish F+ RNA and DNA coliphages, each of the isolated F+ coliphages was subjected to an RNase sensitivity assay. Serially diluted samples of the isolates (undiluted, 10^−2^, 10^−4^, and 10^−6^) were spotted onto 0.8% TSA plates with and without RNase (100 μg mL^−1^). The coliphages that only showed plaques on the RNase-negative plate were considered F+ RNA coliphages and used for further analysis. RNA was extracted using the QIAmp Viral RNA mini kit (Qiagen) to genotype the F+ RNA coliphages. The RT-PCR assay was performed using the QIAGEN One-Step RT-PCR kit (Qiagen) as previously described (35). Amplified RT-PCR products were sequenced by a commercial company, CosmoGene Tech (Seoul, Korea), and each of the nucleic acid sequences was confirmed using NCBI BLAST. To identify the fecal sources using F+ RNA coliphages in the Han River, principal coordinate analysis (PCoA) was performed using Fast UniFrac software (8, 22). Previously identified sequences of F+ RNA coliphages isolated from various animal sources (chicken, cow, goose, pig, swine septic tanks) and human feces were used as a database and compared with nucleic acid sequences of the environmental isolates (20).

In addition to F+ RNA coliphage, the *esp* gene in *E. faecium* has been proposed as a molecular marker of human fecal pollution in environmental waters (28). *Enterococcus* spp. were collected from positive wells of the Enterolert Quanti-tray/2000 using a sterile syringe and needle or sterile pipette tips (17). Membrane-Enterococcus indoxyl-β-d-glucoside agar (mEI) was used to identify and isolate a single *Enterococci* colony at 41°C for 24 h (33). Approximately 20 colonies were chosen from those with blue halos on mEI medium using a sterile wooden toothpick. Each isolate was transferred to a tube containing brain-heart infusion broth (Difco, Detroit, MI, USA) and incubated at 37°C for 16 to 18 h at 180 rpm. After cultivation, the culture was exposed to 96°C for 5 min to extract the DNA (15). The PCR reaction was performed as described in a previous study (28). Briefly, each 25 μL reaction consisted of 2.5 μL template DNA, 10×PCR buffer with 25 mM MgCl_2_ (2.5 μL), 10 mM dNTP mix (1 μL), 0.25 μL of each primer (50 pmol), and 0.25 μL Taq DNA polymerase (Cosmo Genetech Corporation, Seoul, Korea). The PCR conditions were as follows: denaturation at 95°C for 15 min, and 35 cycles of denaturation (94°C for 1 min), annealing (53°C for 1 min), and extension (72°C for 1 min) followed by a final extension at 72°C for 10 min. DNase-/RNase-free water (Qiagen) and DNA extract of *E. faecium* were used as negative and positive controls, respectively, for each PCR run. The size of the expected amplicon was 680 bp and PCR products were analyzed on 1% agarose gels stained with ethidium bromide (Sigma, St. Louis, MO, USA) and visualized under UV illumination.

### Statistical Analysis

Pearson’s correlation coefficient (*r*) was calculated to determine the relationship between rainfall and fecal indicator microorganisms. Accumulated rainfall values for 1 day, 2 days, 3 days, and 4 days were estimated and used as factors to estimate the rainfall effect on microbial contamination. The *t*-test was used to compare the levels of fecal indicator microorganisms in different seasons (summer and winter) and the rainfall condition. Pearson’s correlation coefficients (*r*) between fecal indicator microorganisms and enteric viruses were calculated to characterize the relationships among them. Statistical analyses were performed using SAS software 9.2 (SAS Institute, Cary, NC, USA). Significance was determined at the 95% confidence level.

## Results

### Summary of physical and chemical data

The mean water temperatures of the summer and winter samples were 27.4°C and 5.9°C at S1 and 26.2°C and 9.6°C at S2, respectively. As expected, the water temperature was significantly different between summer and winter (*P*<0.0001) at both sampling sites. The pH value for the S1 samples ranged from 7.5 to 9.4, while that for the S2 samples was approximately 7.3. The turbidity of the S1 and S2 samples ranged from 6 to 60 nephelometric turbidity units (NTU) and from 4 to 73 NTU, respectively (Supplemental material 1). The turbidity of the S2 samples was significantly different before and after rainfall events; however, the pH and temperature did not change following rainfall.

### Concentrations of fecal indicator microorganisms at S1 and S2

Table 1 summarizes the prevalence of fecal indicator microorganisms at S1 and S2 in the Han River. The levels of fecal indicator microorganisms at S2 were higher than at S1 (*P*=0.001). The concentrations of fecal indicator micro-organisms in summer were significantly higher than in winter, particularly at S1. For example, very few or no *Enterococcus* spp. or fecal coliform were found at S1 in winter. The concentrations of total coliform, fecal coliform, and *E. coli* from S1 were significantly higher in summer than during winter (*P*<0.05). Conversely, at S2, no significant differences in any fecal indicator microorganisms were found between summer and winter (*P*>0.05). The concentrations of fecal indicator microorganisms were generally higher at S2 than S1 regardless of season and rainfall.

### The effect of rainfall on the concentrations of fecal indicator microorganisms

Rainfall events substantially increased the levels of fecal indicator microorganisms at both sites (Table 2). The concentrations of total coliform, fecal coliform, and *E. coli* from the S2 site were significantly elevated after rainfall (*P*<0.05). Conversely, no significant differences were found at S1 between before and after rainfall events (*P*>0.05). Table 3 summarizes the correlation between rainfall and fecal indicator microorganisms. Pearson’s correlation was performed between fecal indicator microorganisms and accumulated rainfall (Table 3). For both S1 and S2, three different cumulative rainfall events (1, 2, or 3 days) were significantly correlated with norovirus. At S1, the total coliform, fecal coliform, *E. coli*, and *Enterococcus* spp. were positively correlated with accumulated rainfall for 3 days, while F+ coliphage was positively correlated with accumulated rainfall for 1 day. These data differed slightly from those for S2. Specifically, at S2, the concentrations of fecal coliform were positively correlated with accumulated rainfall for 2 days. In addition, fecal coliform, *E. coli*, and *Enterococcus* spp. showed a strong positive correlation with accumulated rainfall for 1 day in winter samples at S2.

### Characterization of enteric viruses at both sampling sites

Table 3 shows the correlations between rainfall and enteric virus concentrations. Adenovirus was most abundant at both sampling sites. At S1, the level of norovirus (GI and GII) was positively correlated with accumulated 1-day rainfall at S1 (*r*=0.57; *P*=0.03). Norovirus (GI and GII) levels were also correlated with accumulated 1-day rainfall at the S2 site (*r*=0.82; *P*=0.0002); however, no significant correlations between rainfall and adenovirus were found (*P*>0.05). Table 4 summarizes the correlations between enteric virus and fecal indicator microorganisms. The levels of norovirus (GI and GII) were positively correlated with F+ coliphages. In addition, the levels of total coliform, fecal coliform, and F+ coliphages were positively correlated with the levels of enteric viruses.

### Microbial source tracking

**Fig. 2:**
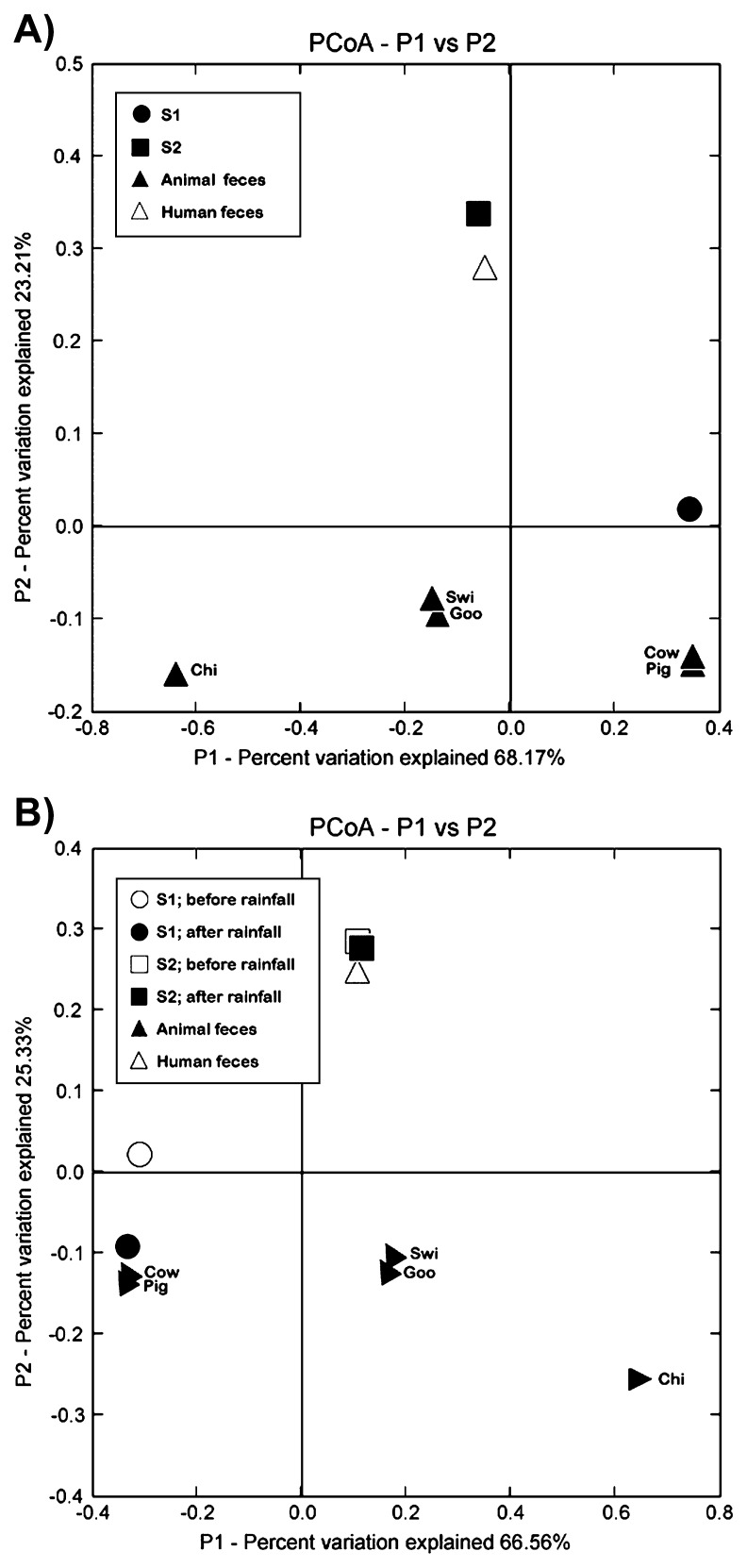
Unifrac unweighted principal coordinate analyses (PCoA) based on two different sampling sites (S1 and S2) (A) and two sampling sites with and without rainfall conditions (B). Letters to the right of ▲ indicate animal fecal sources (chi; chicken, cow; cow, goo; goose, swi; swine septic tank, pig; pig)

Among the 34 collected samples, F+ RNA coliphages were isolated from 12 samples from S1 and 17 samples from S2. Fifty-seven plaques were isolated from S1 samples (Supplemental material 2). Among these, 42 (73.7%) were F+ DNA coliphages and 13 (22.8%) represented genogroup I of the F+ RNA coliphages. Genogroup II of the F+ RNA coliphages was only detected in two (3.5%) summer samples. F+ DNA coliphages were more frequently detected in summer than winter. From the S2 samples, 294 plaques including 168 F+ RNA coliphages were isolated. Among them, 82.7%, 6.5%, and 10.7% of F+ RNA coliphages were categorized into genogroups I, II, and III, respectively. Genogroup I of the F+ RNA coliphages was consistently found throughout the year; however, genogroups II and III of the F+ RNA coliphages were frequently found in winter, but seldom detected in summer. The concentrations of F+ coliphages increased at both the S1 and S2 sites after rainfall (*P*>0.05); however, there was no indication that any specific genogroup of F+ RNA coliphages was associated with rainfall events. To determine the source of fecal contamination using F+ RNA coliphages as described in a previous study, we performed PCoA by comparing 183 nucleic acid sequences of our new isolates with a previously published database of human and animal fecal sources in the same region (Fig. 2) (20). The nucleic acid sequences of F+ RNA coliphages from S2 were very similar to those of human fecal samples (Fig. 2A). On the other hand, the nucleic acid sequences of F+ RNA coliphages from S1 were similar to those from animal fecal sources such as cows and pigs (Fig. 2A). These trends did not change with and without rainfall (Fig. 2B). Following rainfall, the fecal contamination of S1 was even more similar to that from animal sources including cows and pigs. The combined PC1 and PC2 accounted for 91.4% and 91.9% of total variation in the data, respectively (Fig. 2A, 2B).

The *esp* gene, which is a potential marker of human fecal contamination, was only identified in 37 (6.2%) of the 600 isolates of *E. faecium*. The *esp* gene was only identified in one (0.4%) of the 260 *E. faecium* isolates from S1 (Supplemental material 3). Conversely, the *esp* gene was much more frequently found in samples from S2. Specifically, more than 10% of *E. faecium* from S2 exhibited the *esp* gene. The prevalence of the *esp* gene was higher in summer (18.9%) than in winter (1.3%) (*P*=0.09). There were no significant differences in the prevalence of the *esp* gene following rainfall events.

## Discussion

Storm water runoff and heavy rainfall events have been considered important sources of water pollution (4). Here, we demonstrated that rainfall events significantly contributed to fecal contamination in both the upstream and downstream reaches of the Han River. The upstream (S1) and downstream (S2) sites on the Han River had distinct geographic characteristics. The area surrounding the upstream site has been preserved as a drinking water supply for the 20 million people in the metropolitan Seoul area. Various human activities are prohibited and only a few farms are located nearby. Conversely, approximately 10 million people live in the downstream region of the river. The downstream area includes a sewage treatment plant for metropolitan Seoul and is considered a polluted area. Our study demonstrated that rainfall events induce increased fecal contamination in both areas. These results were consistent with those of previous studies (9, 30).

It was found that the levels of fecal indicator micro-organisms and enteric viruses showed strong seasonality. Specifically, much higher concentrations of fecal indicator organisms were found in summer than in winter. For example, the concentrations of both somatic and F+ coliphages increased by 100-fold in summer. Korea experiences a rainy season during the summer. Thus, the increased levels of fecal indicator organisms in the summer months could be caused by 1) an increase in the multiplication of indicator micro-organisms in warmer temperatures and/or 2) the increased rainfall in the summer. Microorganisms tend to multiply better in warmer environments. Unlike bacteria, the viruses showed relatively constant concentrations throughout the year. These results suggest that increased multiplication of fecal bacteria may have occurred during the summer (1). More importantly, heavy rainfall could increase the input of fecal microorganisms from various point and non-point sources into the water. Such occurrences have been well documented in many previous studies (5, 31).

To elucidate the relationship between rainfall and fecal indicator levels, we assessed the accumulated rainfall data. Different aspects of cumulative rainfall data have been applied to predict the levels of fecal microorganisms in water (6, 27). In a previous study, Gersberg *et al.* (6) applied 24 h preceding rainfall data to predict the level of hepatitis A virus and enterovirus in a watershed. Another study by Schiff *et al.* (27) investigated the effect of rainfall on bacterial indicators by categorizing wet/dry days for the previous 72 h period. Here we found that the accumulated rainfall for 1 day or 3 days was the most effective indicator for predicting the levels of fecal microorganisms. While there was no correlation between levels of fecal indicators and rainfall in summer, a strong positive correlation was found in winter. In particular, the levels of fecal coliform, *E. coli*, and *Enterococcus* spp. were correlated with total accumulated rainfall for 1–4 days. It is possible that the weak correlation in summer was due to the summer rainy season in Korea. During the rainy season, there are very frequent rainfall events for at least a month. Throughout this period, the levels of fecal indicator microorganisms were generally high regardless of whether rainfall accumulated for 1 or up to 4 days. For example, the levels of somatic and F+ coliphages with no rain for the previous 3 days during the summer rainy season were 3–10 times higher than those in the non-rainy season (unpublished data). The effect of the rainy season in summer was stronger than the incidence of recent rainfall. Therefore, successive rainfalls for longer periods of time appeared to greatly influence the concentrations of micro-organisms. These results were consistent with a previous study, which reported that correlations between rainfalls and levels of microorganisms differed regionally and seasonally (31).

In this study, F+ DNA coliphages were more frequently observed than F+ RNA coliphages, especially at S1 in summer (Supplemental material 2). Among the F+ RNA coliphages, genogroup I was most frequently found at both the S1 and S2 sites; however, genogroups II and III were also frequently found at S2. These results are consistent with those of previous studies. Specifically, F+ DNA coliphages were commonly detected during warmer months when F+ RNA coliphages were absent from surface water samples (5, 21). Additionally, F+ DNA and F+ RNA genogroup I coliphages were relatively persistent under various environmental conditions in comparison with other bacteriophage groups (21). Higher concentrations of F+ DNA coliphages in summer may be closely related to water temperature and related ecology; however, there are no reports of the associations between F+ DNA coliphages and the origin of fecal sources (20).

Unlike for DNA coliphages, numerous studies have demonstrated that the genogroup of F+ RNA coliphages was closely related to fecal sources. Genogroups II and III of F+ RNA coliphages are mainly associated with human feces. Conversely, genogroups I and IV are mainly related to animal fecal contamination (20, 21, 35); however, some studies have reported that genogroup I coliphages were found in both human and animal feces (29, 32). Our data for F+ RNA coliphages suggest that S1 was mainly contaminated by animal fecal sources, while both animal and human fecal sources contributed to the contamination of the S2 site. These results are consistent with the geographical characteristics of these regions. The upstream (S1) site has been designated as a conservation zone and few people reside in this region. Genogroup I of the F+ RNA coliphages were isolated at the S1 site, which suggests animal fecal contamination (20, 21, 35). Following rainfall, fecal contamination increased, whereas the identified genogroup of F+ RNA coliphages was consistently mostly composed of genogroup I. These data suggest that fecal contamination at S1 was very low when there was no rain, but that it increased, mainly due to animal fecal sources, following rainfall. The sources of this contamination are expected to be mostly of non-point origins, such as farms. Thus, it is expected that the animal-source fecal contamination from these farms is due to runoff during heavy rainfall. Therefore, the major fecal sources at S1 are farmed and wild animals. On the other hand, the downstream sampling site, S2, is located in the middle of Seoul, an area inhabited by approximately 10 million people. There is a sewage treatment plant located near the S2 sampling site, and thus humans are the most likely source of fecal contamination.

In addition to genotyping the F+ RNA coliphages, PCoA was performed using the partial nucleic acid sequences of the F+ RNA bacteriophage for MST in the Han River. Our PCoA results clearly indicated different phylogenetic patterns of bacteriophages isolated from S1 and S2. The results specifically revealed that the upstream (S1) and downstream (S2) sites on the Han River were contaminated by animal and human fecal sources, respectively (Fig. 2A). These results were consistent regardless of rainfall (Fig. 2B).

We performed MST using the *E. faecium esp* gene in addition to F+ RNA coliphages. The *esp* gene was frequently detected at the downstream sampling site of the Han river. The *esp* gene has been used as a human fecal marker despite inconsistent data across studies (3, 15, 17, 28, 36). For example, Scott *et al.* (28) reported that the sensitivity and specificity of the *esp* gene for human fecal contamination were 80–100% and 100%, respectively. Other studies suggested very high specificity of the *esp* gene for human fecal contamination with lower sensitivity (15, 36). Conversely, several studies did not detect any specificity of the *esp* gene toward human fecal contamination (3, 17). In our previous studies, the specificity of the *esp* gene for human fecal contamination was high despite low sensitivity. Our data strongly suggest that fecal contamination at S2 was mainly from human sources whereas human fecal contamination at S1 was very limited.

In conclusion, there was a significant effect of rainfall and the rainy season on increasing animal and human fecal contamination in the Han River. We successfully identified the source of fecal contamination at upstream and downstream sites of the river using F+ RNA coliphages and the *E. faecium esp* gene. Fecal contamination appeared to vary depending on the geographic location, weather, and season; however, the fecal sources were consistent over time.

## Supplementary Material



## Figures and Tables

**Table 1:** Concentrations of fecal indicator microorganisms and enteric viruses at two sampling locations

	S1 (upstream)				S2 (downstream)			
		
	Summer (N=9)		Winter (N=8)		Summer (N=9)		Winter (N=8)	
Total coliform^a^	20,762	(6,800–45,280)^d^	261	(36–3,211)	400,255	(44,260–3,057,000)	231,322	(10,704–1,194,600)
Fecal coliform^a^	842	(149–3,402)^d^	6	(0–263)	56,393	(3,378–542,600)	12,277	(285–120,710)
*E. coli*^a^	238	(30–1,037)^d^	4	(0–54)	32,755	(2,098–398,767)	18,762	(393–203,633)
*Enterococcus* spp.^a^,^c^	238	(20–1,188)	6	(0–74)	3,392	(513–24,695)	2,625	(96–129,970)
Somatic coliphage^a^	12	(2–47)	8	(0–46)	1,343	(285–15,000)	2,460	(147–9,980)
F+ coliphage^a^	7	(0–120)	1	(0–3)	1,358	(158–20,000)	878	(141–3,280)
Adenovirus^b^,^c^	0.519	(0–3.618)	0.362	(0–1.251)	3.852	(0–9.425)	7.328	(0–41.867)
Norovirus^b^,^c^	0.028	(0–0.095)	0	(0)	0.170	(0–0.607)	0.038	(0–0.259)
Astrovirus^b^,^c^	0	(0)	0	(0)	37.977	(0–98.880)	0	(0)
Rotavirus^b^,^c^	0	(0)	0	(0)	0	(0)	0.020	(0–0.157)

a Geometric mean (range) of MPN or PFU 100 mL^−1^

b Mean (range) of viral copies mL^−1^

c Analyzed from 5–8 samples.

d Significant higher than winter (*P*<0.05).

**Table 2:** Levels of fecal indicator microorganisms during rainfall conditions at both S1 and S2

	Rainfall	Total coliform	Fecal coliform	*E. coli*	*Enterococcus* spp.	Somatic coliphage	F+ coliphage
S1 (upstream)	Before rainfall	8,900 (±12,091)^a^	245 (±332)	95 (±152)	17 (±28)	15 (±20)	2 (±4)
	After rainfall	16,330 (±16,271)	1,060 (±1,218)	286 (±349)	377 (±486)	18 (±13)	18 (±39)

S2 (downstream)	Before rainfall	170,607 (±119,344)	16,498 (±13,363)	15,915 (±13,983)	2,301 (±3,802)	2,371 (±4,451)	890 (±763)
	After rainfall	1,247,207 (±1,085,081)	168,490 (±187,709)	139,081 (±142,842)	25,542 (±46,696)	3,308 (±4,738)	4,195 (±6,139)

The levels of fecal indicator microorganisms were calculated by MPN or PFU 100 mL^−1^.

a Mean (±standard deviation)

**Table 3:** Pearson’s correlation between the amount of rainfall and measured microorganisms in this study

		Total coliform	Fecal coliform	*E. coli*	*Enterococcus* spp.	Somatic coliphage	F+ coliphage	Adenovirus	Norovirus	Astrovirus	Rotavirus
S1 (upstream)	Rainfall 1^a^	0.37 (0.15)	**0.55 (0.02)**	0.29 (0.25)	0.55 (0.05)	−0.02 (0.93)	**0.53 (0.03)**	−0.21 (0.47)	**0.57 (0.03)**	n/a^b^	n/a
	Rainfall 2^a^	0.38 (0.14)	**0.53 (0.03)**	0.27 (0.29)	0.53 (0.06)	−0.04 (0.86)	**0.50 (0.04)**	−0.16 (0.59)	0.49 (0.07)	n/a	n/a
	Rainfall 3^a^	**0.71 (0.001)**	**0.71 (0.002)**	**0.77 (0.0003)**	**0.56 (0.05)**	0.29 (0.27)	0.34 (0.19)	−0.32 (0.26)	0.42 (0.14)	n/a	n/a
	Rainfall 4^a^	**0.63 (0.01)**	**0.61 (0.01)**	**0.68 (0.003)**	0.35 (0.25)	0.46 (0.06)	0.32 (0.21)	−0.22 (0.45)	0.42 (0.14)	n/a	n/a

S2 (downstream)	Rainfall 1^a^	0.35 (0.17)	0.46 (0.06)	0.34 (0.19)	0.38 (0.21)	0.31 (0.23)	0.35 (0.17)	−0.12 (0.67)	**0.82 (0.0002)**	−0.07 (0.80)	−0.16 (0.58)
	Rainfall 2^a^	0.38 (0.13)	**0.50 (0.04)**	0.35 (0.16)	0.37 (0.22)	0.34 (0.18)	0.41 (0.10)	−0.14 (0.63)	**0.81 (0.0003)**	−0.07 (0.80)	−0.17 (0.55)
	Rainfall 3^a^	0.26 (0.32)	0.39 (0.13)	0.23 (0.37)	0.25 (0.41)	0.20 (0.44)	0.29 (0.26)	−0.12 (0.66)	**0.83 (0.0001)**	−0.05 (0.86)	−0.17 (0.55)
	Rainfall 4^a^	0.17 (0.53)	0.29 (0.26)	0.13 (0.63)	0.08 (0.81)	0.05 (0.84)	0.21 (0.43)	−0.10 (0.73)	0.48 (0.07)	0.28 (0.32)	−0.19 (0.49)

Both sites	Rainfall 1^a^	0.15 (0.41)	0.22 (0.20)	0.14 (0.42)	0.25 (0.23)	0.11 (0.53)	0.17 (0.35)	−0.14 (0.48)	**0.52 (0.003)**	n/a	n/a
	Rainfall 2^a^	0.15 (0.40)	0.24 (0.18)	0.14 (0.42)	0.22 (0.27)	0.12 (0.20)	0.19 (0.28)	−0.14 (0.46)	**0.49 (0.01)**	n/a	n/a
	Rainfall 3^a^	0.02 (0.89)	0.12 (0.51)	0.02 (0.91)	0.12 (0.55)	−0.02 (0.91)	0.08 (0.67)	−0.20 (0.31)	**0.44 (0.02)**	n/a	n/a
	Rainfall 4^a^	0.01 (0.97)	0.09 (0.61)	−0.01 (0.95)	0.02 (0.91)	−0.07 (0.70)	0.05 (0.77)	−0.15 (0.43)	0.26 (0.17)	n/a	n/a

Bold indicates that the value is statistically significant (*P*<0.05)

a Rainfall 1, 2, 3, and 4 indicate accumulated rainfall for 1 day, 2 days, 3 days, and 4 days, respectively.

b n/a: not available

**Table 4:** Pearson correlation between concentrations of enteric virus and fecal indicator microorganisms

		Total coliform	Fecal coliform	*E. coli*	*Enterococcus* spp.	Somatic coliphage	F+ coliphage
S1 (upstream)	Adenovirus	0.23 (0.43)	−0.22 (0.45)	−0.29 (0.32)	−0.27 (0.42)	−0.36 (0.20)	−0.19 (0.52)
	Norovirus^a^	0.25 (0.40)	0.42 (0.13)	0.17 (0.56)	0.39 (0.24)	0.14 (0.63)	**0.58 (0.03)**
	Any virus^b^	0.24 (0.41)	−0.20 (0.49)	−0.28 (0.33)	−0.25 (0.47)	−0.36 (0.21)	−0.17 (0.57)

S2 (downstream)	Adenovirus	−0.03 (0.90)	−0.06 (0.83)	−0.10 (0.71)	−0.12 (0.69)	0.41 (0.13)	0.04 (0.89)
	Norovirus^a^	−0.04 (0.88)	0.09 (0.76)	−0.02 (0.95)	0.31 (0.30)	0.003 (0.99)	0.01 (0.97)
	Astrovirus	0.29 (0.30)	0.36 (0.19)	0.12 (0.68)	−0.15 (0.63)	−0.01 (0.97)	0.31 (0.25)
	Rotavirus	−0.15 (0.61)	−0.15 (0.60)	−0.11 (0.69)	−0.04 (0.89)	0.40 (0.14)	−0.06 (0.82)
	Any virus^b^	0.26 (0.34)	0.32 (0.24)	0.08 (0.78)	−0.18 (0.55)	0.11 (0.69)	0.31 (0.26)

Both sites	Adenovirus	0.14 (0.45)	0.09 (0.62)	0.07 (0.72)	−0.004 (0.99)	0.50 (0.01)	0.17 (0.39)
	Norovirus^a^	0.13 (0.49)	0.22 (0.26)	0.14 (0.48)	0.37 (0.08)	0.18 (0.34)	0.14 (0.47)
	Any virus^b^	**0.42 (0.02)**	**0.45 (0.01)**	0.26 (0.17)	−0.02 (0.91)	0.32 (0.09)	**0.43 (0.02)**

Bold indicates that the value is statistically significant (*P*<0.05)

a Norovirus: norovirus GI and GII

b Any virus: norovirus GI, GII, adenovirus, astrovirus, and rotavirus.
