# Supplementary material for: Identification of Human and Animal Fecal Contamination after Rainfall in the Han River, Korea
Source: Microbes Environ. 2013 May 11;28(2):187–94. doi: 10.1264/jsme2.ME12187 (PMC4070672; doi:10.1264/jsme2.ME12187)
Supplement: Supplementary file 1 [file 28_187_s1.pdf]

Supplemental material

1. Summary of physical-chemical and rainfall information at both sampling sites

Sampling site	Sampling season	Rainfall (mm)[a]	Rainfall (mm/day)	Temperature[b] (°C)	pH [b]	Turbidity[b] (NTU)
S1	Summer (N=9)	919	16.7	27.4 (2.9)	8.4 (0.7)	21.8 (17.9)
(upstream)	Winter (N=8)	66	0.7	5.9 (3.0)	8.1 (0.5)	9.2 (4.9)
S2	Summer (N=9)	732	13.3	26.2 (2.6)	7.2 (0.2)	30.5 (14.7)
(downstream)	Winter (N=8)	59	0.6	9.6 (3.9)	7.5 (0.3)	27.1 (26.2)

[a] Amount of rainfall during summer (July to September 2010, 55 days) and winter (November 2010 to February 2011, 100 days) season.

[b] Mean (SD)

2. Typing of F+ coliphages isolated from both sampling sites

Sampling site	No. (%) of coliphages					
	F+ RNA coliphage				F+ DNA coliphage	Total
	MS2-like (Group I)	GA-like (Group II)	Qβ-like (Group III)	SP-like (Group IV)		
S1 (upstream)	13 (22.8)	2 (3.5)	0 (0)	0 (0)	42 (73.7)	57 (100)
S2 (downstream)	139 (47.3)	11 (3.7)	18 (6.1)	0 (0)	126 (42.9)	294 (100)
Both sites	152 (43.3)	13 (3.7)	18 (5.1)	0 (0)	168 (47.9)	351 (100)

3. Prevalence of *esp* gene in *E. faecium* isolated from upstream and downstream sites on the Han River

Sampling site	% of *esp* positive in summer	% of *esp* positive in winter	Total (%)
S1 (upstream)	0 (0/160)	1 (1/100)	0.4 (1/260)
S2 (downstream)	18.9 (34/180)	1.3 (2/160)	10.6 (36/340)
Both sites	10 (34/340)	1.2 (3/260)	6.2 (37/600)
